# Phosphorylation of Microtubule- Associated Protein 4 Promotes Hypoxic Endothelial Cell Migration and Proliferation

**DOI:** 10.3389/fphar.2019.00368

**Published:** 2019-04-16

**Authors:** Junhui Zhang, Lingfei Li, Qiong Zhang, Xu Yang, Can Zhang, Xingyue Zhang, Dongxia Zhang, Yanling Lv, Huapei Song, Bing Chen, Yao Liu, Jiongyu Hu, Yuesheng Huang

**Affiliations:** ^1^Institute of Burn Research, Southwest Hospital, Third Military Medical University (Army Medical University), Chongqing, China; ^2^State Key Laboratory of Trauma, Burns and Combined Injury, Southwest Hospital, Third Military Medical University (Army Medical University), Chongqing, China; ^3^Department of Respiratory Medicine, The 983 Hospital of Joint Logistics Support Force of the Chinese People’s Liberation Army, Tianjin, China; ^4^Department of Plastic Surgery, Southwest Hospital, Army Medical University (Third Military Medical University), Chongqing, China; ^5^Endocrinology Department, Southwest Hospital, Third Military Medical University (Army Medical University), Chongqing, China; ^6^Department of Pharmacy, Southwest Hospital, Third Military Medical University (Army Medical University), Chongqing, China

**Keywords:** microtubule, MAP4, p38/MAPK, cell migration, proliferation

## Abstract

Endothelial cells play a critical role in the process of angiogenesis during skin wound healing. The migration and proliferation of endothelial cells are processes that are initiated by the hypoxic microenvironment in a wound, but the underlying mechanisms remain largely unknown. Here, we identified a novel role for microtubule-associated protein 4 (MAP4) in angiogenesis. We firstly demonstrated that MAP4 phosphorylation was induced in hypoxic endothelial cells; the increase in MAP4 phosphorylation enhanced the migration and proliferation of endothelial cells. We also found that hypoxia (2% O_2_) activated p38/mitogen-activated protein kinase (MAPK) signaling, and we identified p38/MAPK as an upstream regulator of MAP4 phosphorylation in endothelial cells. Moreover, we showed that the promigration and proproliferation effects of MAP4 phosphorylation were attributed to its role in microtubule dynamics. These results indicated that MAP4 phosphorylation induced by p38/MAPK signaling promotes angiogenesis by inducing the proliferation and migration of endothelial cells cultured under hypoxic conditions via microtubule dynamics regulation. These findings provide new insights into the potential mechanisms underlying the initiation of the migration and proliferation of endothelial cells.

## Introduction

Endothelial cell (EC) proliferation and migration are essential processes during wound healing, due to their roles in angiogenesis (Munoz-Chapuli et al., 2004; Lamalice et al., 2007; Banyard et al., 2016), which is required to restore oxygen and nutrient supplies (DiPietro, 2013). However, if angiogenesis is deregulated, wounds may not heal. Upon wounding, hypoxia occurs at the wound edge due to the interrupted oxygen supply and excessive oxygen consumption (Falanga, 1988; O’Toole et al., 1997; Yip, 2015). The hypoxic microenvironment has been proven to be an important initial factor for the healing skin wounds, but the underlying mechanisms remain largely unknown.

Microtubules (MTs) are one of the major components of the eukaryotic cytoskeleton, and they are composed of α and β-tubulin heterodimers; they have a variety of biological functions, such as mediating cell mitosis, cell movement, and integrating transmembrane signals in many eukaryotic cells, including ECs (Liu et al., 1998; Kreitzer and Myat, 2018; LaFlamme et al., 2018). The transition between the polymerization and depolymerization of α- and β-tubulin is defined as MT dynamics, which is regulated by destabilizing proteins (e.g., stathmin family) (Cassimeris, 2002; Giampietro et al., 2005) and stabilizing proteins, such as microtubule-associated proteins (MAPs) (Kremer et al., 2005). MAP4, the major MAP protein expressed in non-neuronal mammalian cells, is capable of regulating MT assembly and stability both *in vitro* and *in vivo*. The phosphorylation of MAP4 impairs its function and induces its detachment from MTs, resulting in the depolymerization of MTs. The crucial phosphorylation sites in human MAP4 responsible for altering its function are S768 and S787 (Srsen et al., 1999; Kitazawa et al., 2000; Hu et al., 2010). It has been reported that MAP4 phosphorylation affects cell cycle progression (Chang et al., 2001) and apoptosis (Hu et al., 2014). MAP4 was recently shown to promote cell invasion in the progression of solid tumors (Ou et al., 2014; Jiang et al., 2016; Xia et al., 2018). To date, the contributions of MAP4 phosphorylation to the proliferation and migration of ECs during angiogenesis remain largely unknown.

Previously, we reported that p38/mitogen-activated protein kinase (MAPK) induces MAP4 phosphorylation and MT disassembly in hypoxic cardiomyocytes (Hu et al., 2014). In addition, p38/MAPK is an important kinase required for the cytoskeletal reorganization that precedes the migration and proliferation of various cell types (Huang et al., 2004). Therefore, MAP4 phosphorylation controlled by p38/MAPK may regulate MT dynamics, migration, and proliferation in ECs.

Hypoxic stress after acute injuries stimulates gene expression and growth factor synthesis, contributing to wound angiogenesis (Yip, 2015). Here, we reported that MAP4 phosphorylation promoted the migration and proliferation of ECs subjected to short-term hypoxia (2% O_2_). Additionally, MAP4 phosphorylation was induced in response to hypoxia through the activation of p38/MAPK signaling. Mechanistically, MAP4 phosphorylation increased cell migration and proliferation by mediating MTs dynamics in ECs. Together, our data suggested a novel role for MAP4 phosphorylation in angiogenesis.

## Results

### Hypoxia Promotes the Migration and Proliferation of Endothelial Cells (ECs)

**FIGURE 1:**
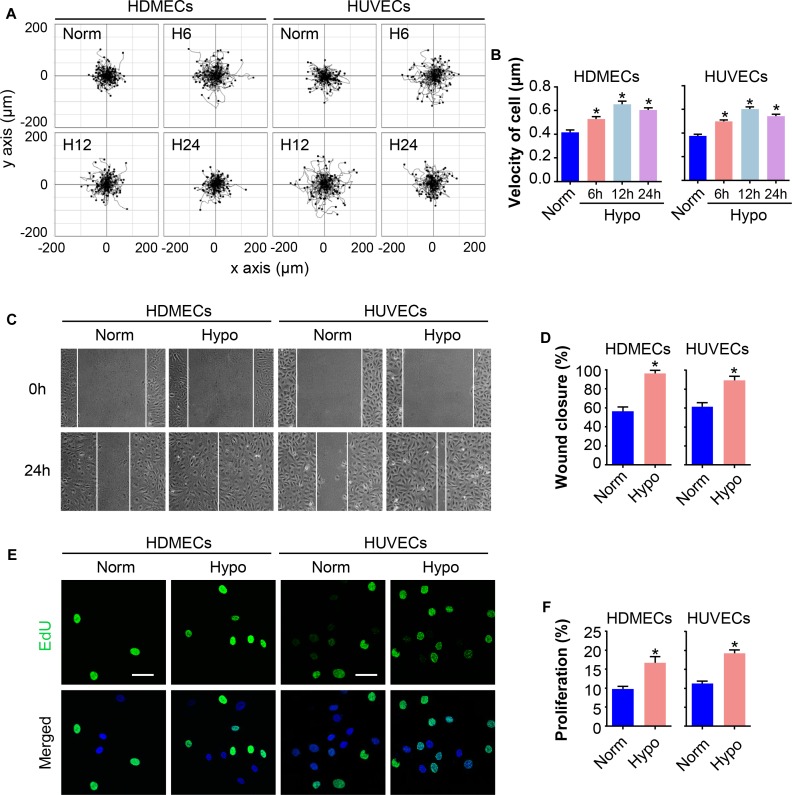
Hypoxia promotes EC migration and proliferation. HDMECs and HUVECs were exposed to hypoxia (2% O_2_) and incubated for the indicated times to investigate the migration and proliferation of ECs under hypoxia stress. **(A)** Single cell motility assays were performed to detect the motility of the indicated cells (*n* = 5). Representative images of cell trajectories are shown. **(B)** Graph quantifying the average velocity of cell movement. The results are presented as the means ± SEM. **(C)** Scratch wound healing assays were performed to detect the migration of the indicated cells. Images of the scratch wound were captured after 24 h of culture with or without the hypoxia treatment (*n* = 5). Representative images of the wound healing assay are shown. Scale bar = 100 μm. **(D)** Graph quantifying the rate of wound closure. The results are presented as the means ± SEM. **(E)** After culture in the presence or absence of hypoxia for 24 h, the indicated cells were subjected to EdU staining to assess cell proliferation (*n* = 5). Representative images of EdU staining (green) in cultured cells. Nuclei were stained with 4′, 6-diamidino-2-phenylindole (DAPI, blue). Scale bar = 50 μm. **(F)** Graph quantifying the number of EdU-positive cells in **(E)**. Data are presented as the means ± SEM. ^∗^*P* < 0.05 compared with the Norm group. Norm, normoxia; Hypo, hypoxia.

Migration and proliferation of ECs are critical processes for angiogenesis during wound repair (DiPietro, 2013). Human umbilical vein endothelial cells (HUVECs) and human dermal microvascular endothelial cells (HDMECs) were subjected to hypoxia for 6, 12, or 24 h to determine the effect of hypoxia on ECs. The motility of single cells was assessed. Remarkably, hypoxia enhanced cell motility in a time-dependent manner, with the maximum effect achieved after treatment with 2% O_2_ for 12 h, as shown by a larger range of cell trajectories and greater velocity of cell movement (Figure 1A,B). Then, a scratch wound healing assay was performed to evaluate the migratory activity of the cells cultured under hypoxia (2% O_2_ for 12 h). As expected, the migration of hypoxic HDMECs and HUVECs was significantly increased compared to normoxic cells, as shown by the increased percentage of gap closure after wounding (Figure 1C,D). In addition, EdU staining, an advanced method for assessing cell proliferation, revealed a marked increase in the proliferation of HDMECs and HUVECs cultured under hypoxia, as indicated by the positive staining for of 5-ethynyl-2′-deoxyuridine (EdU, green) in cell nuclei (Figure 1E,F). Thus, the migration and proliferation of ECs were indeed enhanced under hypoxic conditions.

### MAP4 Phosphorylation Is Increased in Hypoxic ECs

**FIGURE 2:**
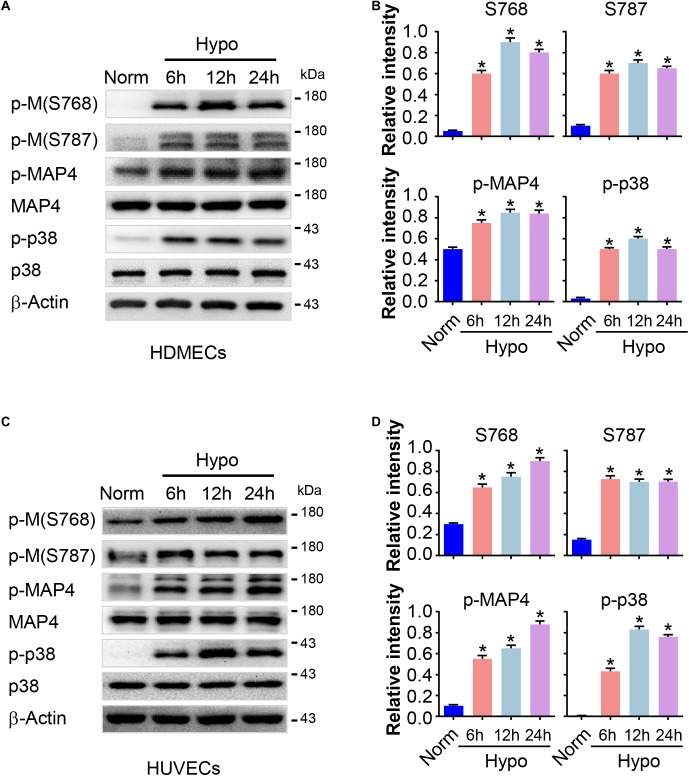
MAP4 phosphorylation is increased in hypoxic ECs. HDMECs and HUVECs were subjected to hypoxia (2% O_2_) and incubated for the indicated times (0, 6, 12, and 24 h). Protein extracts were analyzed using Western blotting to determine the levels of MAP4 phosphorylation and the activity of p38/MAPK (*n* = 5). **(A,C)** Representative Western blots are shown. β-Actin was used as a loading control. Data are presented as the means ± SEM. **(B,D)** The graph presents the means ± SEM of the relative integrated signals. ^∗^*P* < 0.05 compared with the Norm group. p-M, p-MAP4.

Previously, we have revealed an increase in MAP4 phosphorylation in hypoxic cardiomyocytes (Hu et al., 2014). MAP4 phosphorylation was analyzed in HDMECs and HUVECs treated with or without hypoxia using Western blotting to investigate the potential effects of MAP4 phosphorylation on EC proliferation and migration. As shown in Figure 2A,B, low basal levels of MAP4 phosphorylated on S768 and S787 were observed in HDMECs cultured under normoxic condition. However, a marked increase in the levels of phosphorylation at both residues and p-MAP4 was induced in a time-dependent manner in response to hypoxia (2% O_2_), with MAP4 levels unchanged. Meanwhile, we analyzed the activation of p38/MAPK signaling, which was reported to control the phosphorylation of MAP4 in other contexts (Li et al., 2015, 2018). The activity of the p38/MAPK signaling pathway was low under normoxic conditions, while the hypoxia treatment significantly increased the activation of the p38/MAPK signaling pathway, as determined with a phospho-specific p38/MAPK antibody (Thr180/Tyr182). Furthermore, the observations that the hypoxia treatment increased MAP4 phosphorylation and the activation of p38/MAPK signaling were confirmed in HUVECs (Figure 2C,D).

### MAP4 Phosphorylation Regulates EC Migration and Proliferation

**FIGURE 3:**
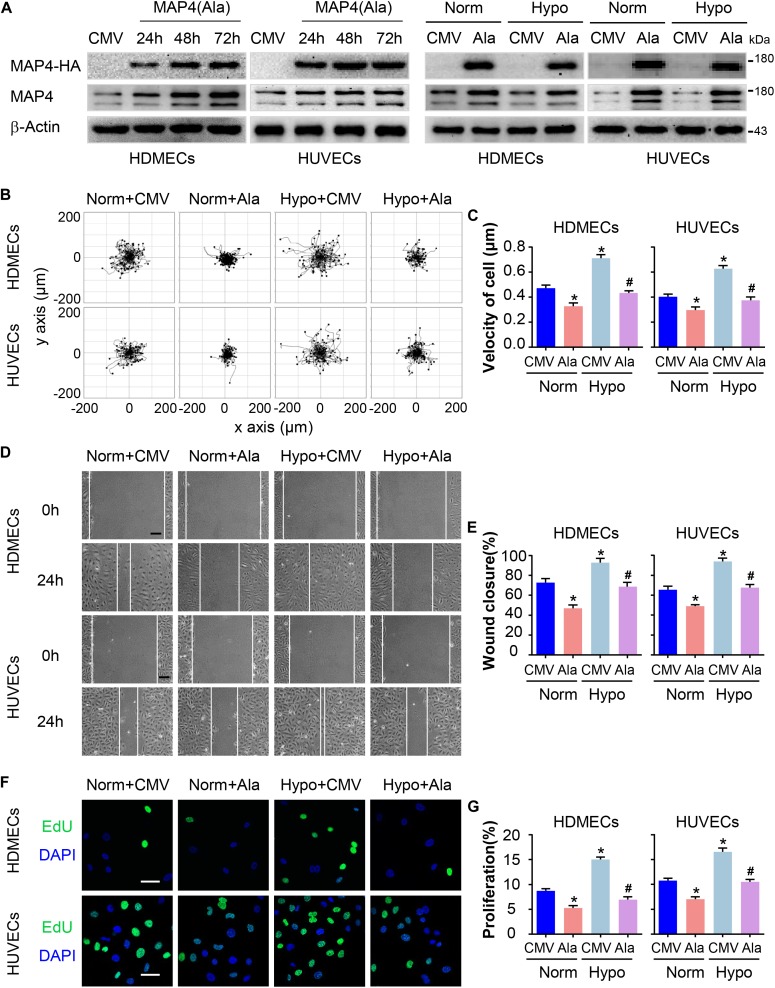
MAP4 phosphorylation regulates EC migration and proliferation. **(A)** Total protein extracts from cells transfected with the MAP4(Ala) adenovirus were analyzed using Western blotting to confirm that the adenovirus was transfected at comparable levels in HDMECs and HUVECs (*n* = 5). Then, cells were subjected to hypoxia stress after transfection with CMV-null or MAP4(Ala) for 48 h. β-Actin was used as the loading control. **(B)** Single cell motility assays were performed to detect the motility of the indicated cells (*n* = 5). Representative images of cell trajectories are shown. **(C)** Graph quantifying the average velocity of cell movement. The results are presented as the means ± SEM. **(D)** Scratch wound healing assays were performed to detect the migration of the indicated cells (*n* = 5). Images of the scratch wounds were captured after 24 h of culture in the presence or absence of hypoxia. Representative images of the wound healing assay are shown. Scale bar = 100 μm. **(E)** Graph quantifying the rate of wound closure. The results are presented as the means ± SEM. **(F)** After transfection with the indicated adenovirus for 48 h, the proliferation of the indicated cells was evaluated using EdU staining. Representative images of EdU staining (green) in cultured cells are shown (*n* = 5). Scale bar = 50 μm. Nuclei were stained with DAPI (blue). **(G)** Graph quantifying the number of EdU-positive cells in **(E)**. Data are presented as the means ± SEM. ^∗^*P* < 0.05 compared with the Norm + CMV group and ^#^*P* < 0.05 compared with the Hypo + CMV group. CMV, CMV-null; Ala, MAP4(Ala); MKK6, MKK6(Glu).

A MAP4(Ala) mutant was constructed by changing S768 and S787 residues to alanines [MAP4(Ala)] to mimic the non-phosphorylated forms and to elucidate whether the phosphorylation status of MAP4 was involved in EC migration and proliferation. HA-tagged MAP4(Ala) or CMV-null was overexpressed at comparable levels in both HDMECs and HUVECs, as determined by Western blot analysis (Figure 3A). Then, we transfected the MAP4(Ala) mutant or CMV-null into HDMECs and HUVECs prior to the hypoxia treatment (Figure 3A). Consequently, changes in cell migration were determined using two different assays: a single cell motility assay and a scratch wound healing assay. As expected, the migratory capacity of HDMECs and HUVECs in the hypoxic CMV-null group was significantly greater than the normoxic CMV-null group, whereas the migratory capacity was dramatically decreased in MAP4(Ala) transfectants compared with cells transfected with CMV-null under hypoxia stress (Figure 3B–E). Similarly, the increased proliferation of HDMECs and HUVECs subjected to hypoxia was markedly reduced in MAP4(Ala) transfectants compared with cells transfected with CMV-null, as depicted using EdU staining (Figure 3F,G). Based on these observations, MAP4 phosphorylation indeed promoted the migration and proliferation of ECs.

### P38/MAPK Activation Mediates MAP4 Phosphorylation and the Migration and Proliferation of Hypoxic ECs

**FIGURE 4:**
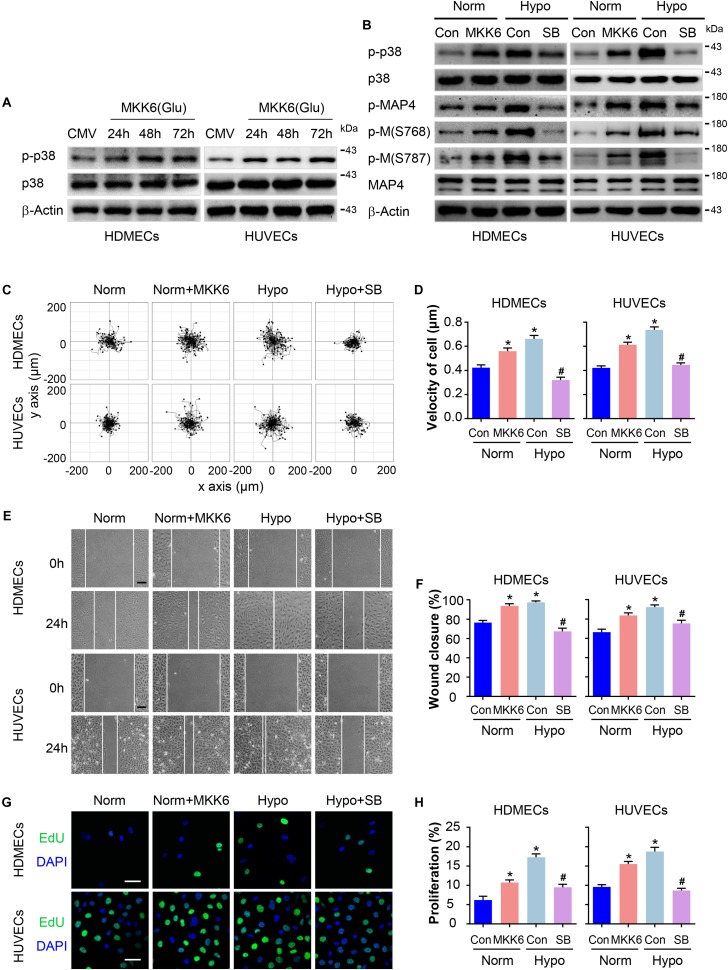
P38/MAPK activation mediates MAP4 phosphorylation and the migration and proliferation of hypoxic ECs. **(A)** Confirmation of comparable levels of adenovirus transfection in ECs. Total protein extracts obtained from cells transfected with the MKK6(Glu) adenovirus were analyzed using Western blotting (*n* = 5). β-Actin was used as a loading control. **(B)** HDMECs and HUVECs were transfected with the MKK6(Glu) adenovirus and cultured under normoxic conditions or exposed to a specific p38/MAPK inhibitor SB (5 μM) prior to the hypoxia treatment. Western blot showing p38/MAPK activity and MAP4 phosphorylation in cells subjected to the indicated treatments (*n* = 5). β-Actin was used as the loading control. **(C)** Representative images of cell trajectories from the single cell motility assays are shown (*n* = 5). **(D)** Graph quantifying the average velocity of cell movement. The results are presented as the means ± SEM. **(E)** Representative images of the indicated groups from scratch wound healing assays are shown (*n* = 5). Scale bar = 100 μm. **(F)** Graph quantifying the rate of wound closure. The results are presented as the means ± SEM. **(G)** Representative images of EdU staining (green) in cultured cells are shown (*n* = 5). Scale bar = 50 μm. Nuclei were stained with DAPI (blue). **(H)** Graph quantifying the number of EdU-positive cells in **(G)**. Data are presented as the means ± SEM. ^∗^*P* < 0.05 compared with the Norm + Con group and ^#^*P* < 0.05 compared with the Hypo + Con group.

We have previously identified MAP4 phosphorylation as a downstream target of p38/MAPK signaling in hypoxic cardiomyocytes and LPS-stimulated human pulmonary microvascular endothelial cells (HPMECs) (Hu et al., 2014; Li et al., 2015). In the present study, p38/MAPK activation and MAP4 phosphorylation were consistently increased in hypoxic HDMECs and HUVECs (Figure 2). We constructed a MKK6(Glu) adenovirus to persistently activate MKK6, which induced p38/MAPK activation, and it was used as the positive control to explore whether the p38/MAPK signaling is involved in hypoxia-induced MAP4 phosphorylation, migration, and proliferation in ECs (Figure 4A). Conversely, the specific p38/MAPK inhibitor SB203580 (SB, 5 μM) was used to inhibit the p38/MAPK signaling pathway in HDMECs and HUVECs under hypoxia (2% O_2_). MAP4 phosphorylation (p-MAP4, S737, and S760) in the MKK6(Glu) group was significantly increased, and SB (5 μM) decreased MAP4 phosphorylation induced by the hypoxia treatment (Figure 4B). Next, we detected the influence of p38/MAPK activation on EC migration and proliferation. Our observations indicated that the MKK6(Glu) group exhibited a remarkably increased range of cell trajectories and velocity of cell movement in HDMECs and HUVECs cultured under normoxic conditions, while the p38/MAPK inhibitor SB inhibited the hypoxia-induced cell motility (Figure 4C,D). Using the scratch wound healing assay, MKK6(Glu) transfection increased the percentage of gap closure after wounding, while SB significantly delayed the migration of hypoxic HDMECs and HUVECs (Figure 4E,F). In addition, the results of EdU staining, which was applied to assess cell proliferation, demonstrated that MKK6(Glu) transfection induced cell proliferation with a higher percentage of EdU-positive HDMECs and HUVECs cultured under normoxic conditions, while the p38/MAPK inhibitor remarkably reduced the proliferation of hypoxic HDMECs and HUVECs (Figure 4G,H). Thus, p38/MAPK was a critical upstream regulator of MAP4 phosphorylation and induced the migration and proliferation of HDMECs and HUVECs under hypoxic stress.

### MAP4 Phosphorylation Plays a Role in p38/MAPK Signaling-Induced EC Migration and Proliferation

**FIGURE 5:**
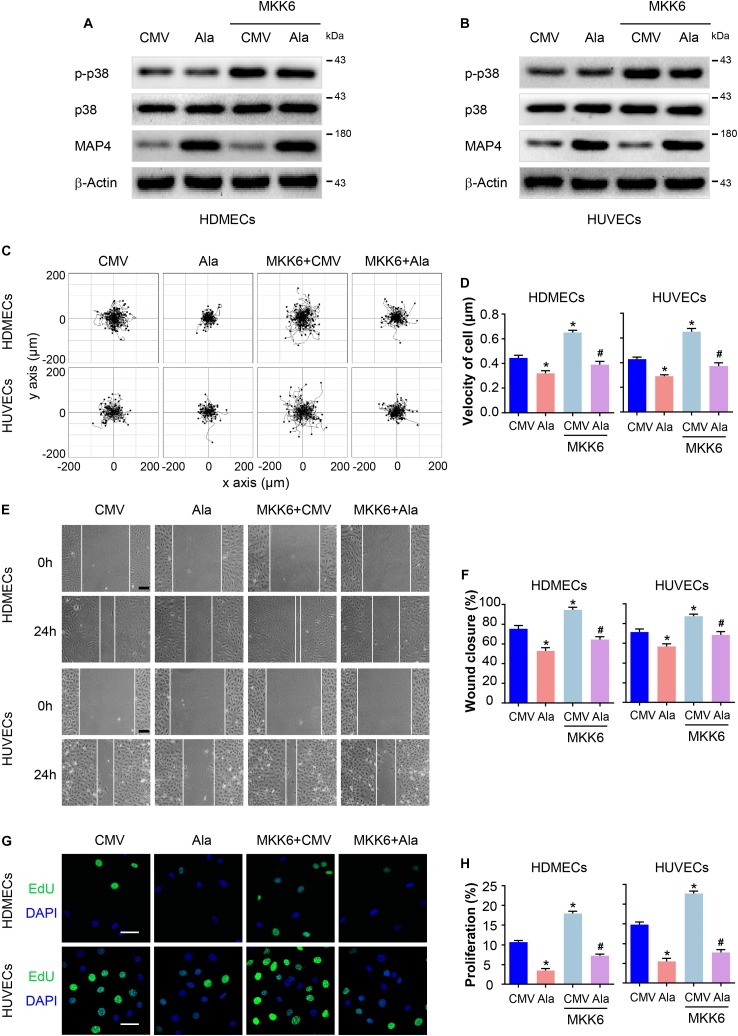
MAP4 phosphorylation plays a role in p38/MAPK signaling-induced EC migration and proliferation. HDMECs and HUVECs were transfected with MAP4(Ala), MKK6(Glu) or both constructs to determine the role of p38/MAPK signaling in MAP4 phosphorylation-induced EC migration and proliferation. Western blots were performed to analyze the activities of p38/MAPK and the levels of MAP4 in HDMECs **(A)** and HUVECs **(B)** (*n* = 5). Single cell motility and scratch wound healing assays were performed to detect cell migration (*n* = 5). **(C)** Representative images of cell trajectories are shown. **(D)** Graph quantifying the average velocity of cell movement. The results are presented as the means ± SEM. **(E)** Representative images of the indicated groups are shown. Scale bar = 100 μm. **(F)** Graph quantifying the rate of wound closure. The results are presented as the means ± SEM. **(G)** Representative images of EdU staining (green) in cultured cells are shown. Nuclei were stained with DAPI (blue) (*n* = 5). Scale bar = 50 μm. **(H)** Graph quantifying the number of EdU-positive cells in **(G)**. Data are presented as the means ± SEM. ^∗^*P* < 0.05 compared with the CMV group and ^#^*P* < 0.05 compared with the MKK6 + CMV group.

Furthermore, we determined whether MAP4 phosphorylation was involved in p38/MAPK -mediated EC migration and proliferation in response to hypoxia. HDMECs and HUVECs were transiently transfected with MAP4(Ala), MKK6(Glu), or both constructs. MKK6(Glu) transfection notably increased p38/MAPK activation, which was not influenced by MAP4(Ala) and MKK6(Glu) cotransfection, as determined using Western blotting (Figure 5A,B), further confirming that p38/MAPK was the upstream kinase responsible for MAP4 phosphorylation and the observations described above. As expected, MKK6(Glu) increased the range of cell trajectories and the velocity of cell movement. Interestingly, MAP4(Ala) overexpression significantly abolished the p38/MAPK-induced cell motility (Figure 5C,D). In the scratch wound healing assay, MAP4(Ala) suppressed the migration of HDMECs and HUVECs induced by MKK6(Glu) transfection (Figure 5E,F). In addition, MAP4(Ala) overexpression significantly abrogated the MKK6(Glu)-induced cell proliferation, as depicted by a decrease in the percentage of EdU-positive HDMECs and HUVECs (Figure 5G,H). Taken together, these observations denoted an essential role for MAP4 phosphorylation in p38/MAPK-induced EC migration and proliferation.

### The p38/MAPK Signaling Pathway Involved in the MAP4 Phosphorylation- Mediated MT Depolymerization in Hypoxic ECs

**FIGURE 6:**
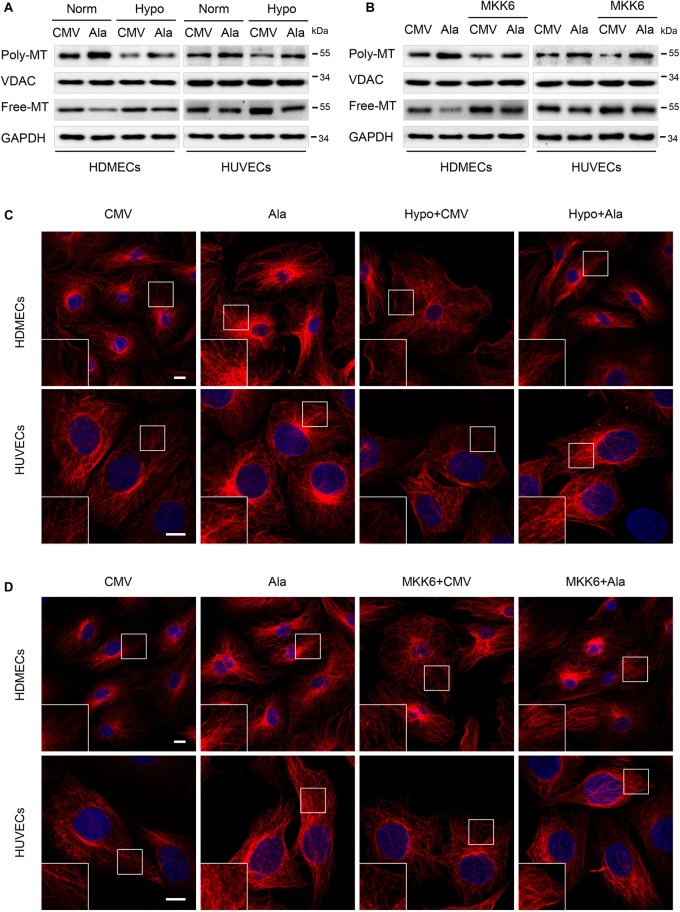
The p38/MAPK signaling involved in the MAP4 phosphorylation-mediated MT depolymerization in hypoxic ECs. HDMECs and HUVECs were transfected with MAP4(Ala), MKK6(Glu) or both constructs and cultured for 48 h before being exposed to hypoxia. **(A,B)** Western blots were performed to analyze the polymerized and free tubulin levels; VDAC and GAPDH were used as the loading controls for these fractions (*n* = 5). **(C,D)** Representative images of stained MT in the indicated keratinocytes are shown. The boxed areas are shown at higher magnification in the inserts to illustrate details (*n* = 5). Scale bar = 10 μm.

Previously, the balance between MAP4 phosphorylation and dephosphorylation has been shown to be an essential factor that controls microtubule (MT) assembly and stabilization. Once phosphorylated, MAP4 dissociates from MTs, leading to MT disruption (Ramkumar et al., 2018). MTs have fundamental roles in many essential biological processes ranging from intracellular trafficking and the positioning of cellular components in interphase to the formation of mitotic spindle during cell proliferation and the maintenance of cell morphology and cell motility (Kaverina and Straube, 2011; Akhmanova and Steinmetz, 2015; Steinmetz and Prota, 2018). Thus, we analyzed the roles of p38/MAPK signaling and MAP4 phosphorylation in MT depolymerization in hypoxic HDMECs and HUVECs. The MAP4(Ala) mutant was overexpressed in the two EC lines prior to the hypoxia treatment. Western blots showed that MAP4(Ala) transfection reversed the reduced levels of polymerized tubulin and increased the levels of free tubulin in hypoxic cells (Figure 6A). The MT morphology in the indicated cells was observed using a confocal microscope (Figure 6C). In HDMECs and HUVECs, the MTs of CMV-null group were well-organized and displayed a faint, uniformly distributed lattice network, whereas the cells under hypoxia stress showed clear signs of MT disruption and modification, as manifested by a less regular organization and some breakages along the edges of the cell or in the vicinity of the nuclei. Thus, the MTs were depolymerized. After transfection with MAP4(Ala), MT disruption was suppressed and reversed. These data indicated that MAP4(Ala) protected ECs from hypoxia-induced MT depolymerization.

P38/MAPK is an important regulator of MT dynamics, which are involved in many different cellular events (Hu et al., 2014; Li et al., 2015; You et al., 2016). The finding that p38/MAPK regulated MAP4 phosphorylation, which has been shown to control MT stabilization in ECs, motivated us to assess the role of MAP4 phosphorylation in p38/MAPK-induced MT dynamics. ECs overexpressing MAP4(Ala) were significantly more resistant than the CMV-null control ECs to MT disassembly in response to MKK6(Glu) transfection, as assessed using MT immunofluorescence staining and a tubulin fraction analysis (Figure 6B,D). These findings were consistent with our concept of a critical role for MAP4 phosphorylation in p38/MAPK-mediated MT depolymerization in hypoxic ECs.

## Discussion

**FIGURE 7:**
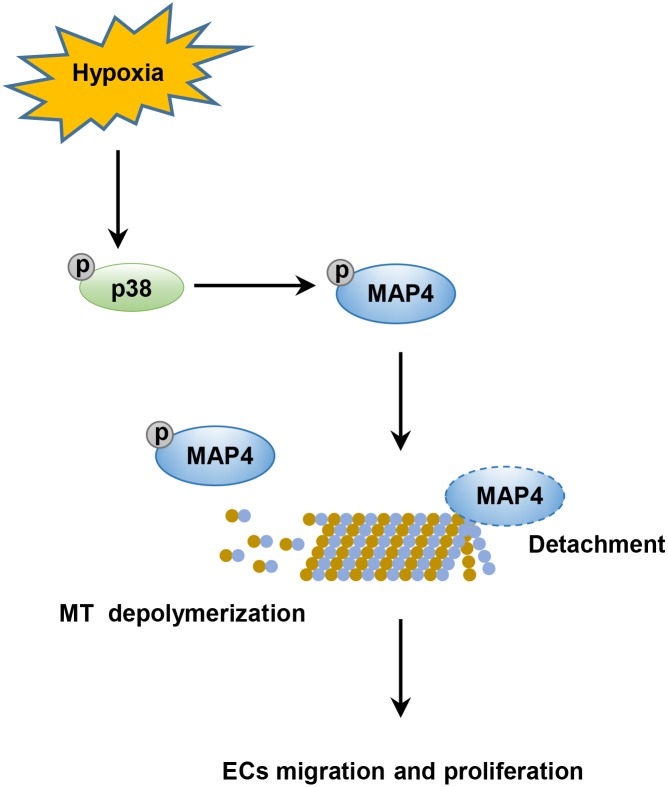
Schematic illustrating that phosphorylated MAP4 promotes hypoxic endothelial cell migration and proliferation. The hypoxic environment stimulates the activation of the p38/MAPK signaling pathway and consequently induces the phosphorylation of MAP4 at S768 and S787. Once phosphorylated, MAP4 detaches from MTs; this results in the depolymerization of MTs, which is required for the migration and proliferation of ECs.

Angiogenesis is a complex process that requires the coordinated migration and proliferation of ECs to form a lumen-containing tubular vessel with blood flow. Here, we analyzed the role of MAP4 phosphorylation-mediated microtubule disassembly in EC migration and proliferation. The salient findings presented here revealed that MAP4 phosphorylation (S768 and S787)-dependent MT disassembly was required for EC migration and proliferation, as determined in HDMECs and HUVECs. Moreover, our data demonstrated that hypoxia upregulated MAP4 phosphorylation through the activation of p38/MAPK signaling in ECs, which contributed to MT disassembly, and subsequently enhanced cell migration and proliferation (Figure 7).

Hypoxia stimulates a variety of intracellular signaling pathways that regulate the proliferation, migration, and survival of various cell types (Xing et al., 2011; Joseph et al., 2015; Nagaraju et al., 2015). Previously, the early use of semi-occlusive dressings (inducing a hypoxic microenvironment) after wounding promoted wound repair compared with wounds that were allowed to air dry (Hopf and Rollins, 2007), but the precise mechanisms were still incompletely elucidated. Some investigators have mainly focused on keratinocyte migration under hypoxia stress (O’Toole et al., 1997; Zhang et al., 2017). To date, the role of the wound hypoxic microenvironment in the biological behavior of vascular ECs has received little attention. As one of the major cell types that participate in wound repair, ECs proliferate from the original vascular bed and migrate to the wound bed to produce a new capillary network, eventually reconstructing the blood circulation to replenish oxygen and energetic substances for re-epithelialization. Based on the *ex vivo* results from the present study, hypoxia (2% O_2_) enhanced the migration and proliferation of HDMECs and HUVECs. These observations provide a novel mechanism for the early hypoxia-induced wound repair from the perspective of vascular ECs.

MAP4 is mainly expressed in non-neuronal tissues and is ubiquitously present in all cell types (Parysek et al., 1984). The phosphorylation of the serine sites in its microtubule-binding domain regulates microtubule dynamics (Srsen et al., 1999; Kitazawa et al., 2000; Chang et al., 2001; Hu et al., 2014). Altered levels of MAP4 phosphorylation affect cell cycle progression (Ookata et al., 1995; Ramkumar et al., 2018). Recently, some studies have reported a role for MAP4 in human cancers. For instance, the cAMP/PKA pathway is involved in bladder cancer cell invasion by targeting MAP4-dependent MT dynamics (Ou et al., 2014). Overexpression of MAP4 is associated with a poor prognosis of esophageal squamous cell carcinoma and promotes cell invasion and migration through the MAP4-ERK-Jun-VEGF signaling pathway (Jiang et al., 2016). MAP4 knockdown efficiently suppresses lung adenocarcinoma cell migration and invasion (Xia et al., 2018). Moreover, the microtubule destabilizer stathmin promotes the epithelial–mesenchymal transition (EMT) and metastasis of tumor cells by reducing microtubule polymerization (Li et al., 2011), which validates the role of microtubule dynamics in cell migration. Consistent with these findings, the inhibition of MAP4 phosphorylation notably suppressed the hypoxia-induced EC migration and proliferation through MT disassembly in the present study. These results indicate a promigration and proproliferation effect of MAP4 phosphorylation mediated by regulating microtubule dynamics.

Mitogen-activated protein kinases are important regulators of signal transduction upon exposure to stress-induced stimuli or extracellular growth factors and are crucial for the control of numerous cellular processes, including proliferation, migration and survival (Kim and Choi, 2010). The p38/MAPK signaling pathway is activated in multiple cells under hypoxic conditions (Hirota et al., 2015; Park and Rongo, 2016; Chen et al., 2018), and MKK6 is recognized as a specific upstream regulator of p38/MAPK signaling (Stramucci et al., 2018). As shown in our previous studies, p38/MAPK was upregulated through ROS generation in keratinocytes under hypoxia stress (Zhang et al., 2019). Meanwhile, the activation of the p38/MAPK pathway is implicated in MAP4 phosphorylation in hypoxic cardiomyocytes (Hu et al., 2014; Li et al., 2018). We investigated the effect of p38/MAPK signaling on MAP4 phosphorylation to obtain further insights into the phosphorylation of MAP4 in ECs under hypoxia. Consistently, the specific inhibitor SB203580 and the endogenous activator of p38/MAPK MKK6(Glu) confirmed that the p38/MAPK pathway was required for the hypoxia-induced migration and proliferation of ECs by inducing MAP4 phosphorylation and MT disassembly.

In summary, our current work reveals a novel mechanism underlying the hypoxia-induced migration and proliferation of ECs. MAP4 phosphorylation controlled by p38/MAPK signaling under hypoxia promotes the migration and proliferation of ECs by inducing MT disassembly. Furthermore, our results provide a potential target for development of new treatments for angiogenesis.

## Materials and Methods

### Cell Culture

Human dermal microvascular endothelial cells were obtained from ScienCell Research Laboratories (San Diego, CA, United States) and cultured in endothelial cell medium (ECM) supplemented with 5% fetal bovine serum (FBS, ScienCell Research Laboratories), 1% endothelial cell growth supplement (ECGS, ScienCell Research Laboratories), and a 1% penicillin/streptomycin (P/S) solution (ScienCell Research Laboratories, 100 U/ml penicillin and 100 μg/ml streptomycin). HUVECs were obtained from the American Type Culture Collection (ATCC, United States) and cultured in RPMI 1640 medium (SH30809, HyClone) containing 10% FBS (10100139, Gibco), 100 U/ml penicillin, and 100 μg/ml streptomycin (Beyotime, China). The cells were incubated at 37°C in a 5% CO_2_ atmosphere with 95% humidity.

### Hypoxia Exposure

Hypoxic conditions of 2% O_2_, 5% CO_2_, and 93% N_2_ were maintained by providing a constant flow of nitrogen using a Forma Series II Water Jacket CO_2_ incubator (model: 3131; Thermo Scientific), which maintained the desired temperature (37°C) and O_2_ level. A p38 inhibitor (SB203580 (SB), Selleck, 5 μM) was added to the medium and incubated with the cells at 37°C for 30 min before the hypoxia treatment.

### Scratch Wound Healing Assay and Quantitative Analysis

Monolayers of cells plated in 6-well plates were scratched with a 10-μl plastic pipette tip an incubation at 37°C for 2 h with mitomycin-C (S8146, Selleck, final concentration: 5 μg/mL) to inhibit cell proliferation, and then rinsed with medium to remove any cell debris (Zhang et al., 2017). The wound healing process was monitored with an inverted light microscope (Olympus, Japan). The cellular migratory capacity was defined as the rate of wound closure (%), which was analyzed using NIH ImageJ software^1^.

### Single Cell Motility Assay and Quantitative Analysis

Cells were seeded into 24-well plates at a density of 0.5 × 10^4^ cells/cm^2^ in the corresponding medium. Then, time- lapse imaging was performed with a Zeiss imaging system (Carl Zeiss Meditec, Jena, Germany), which was equipped with a CO_2_- and temperature-controlled chamber. Images were acquired every 10 min for 6 h. Later, cell trajectories were obtained by tracing the position of the cell nucleus at frame intervals of 6 min using NIH ImageJ software, and the velocity (μm/min) of each cell was defined as the total length (μm) of the trajectories divided by the time (min), which reflected cell motility.

### Site-Directed Mutagenesis of MAP4 and MKK6, Recombinant Adenovirus Construction and Transduction

Primers were designed to generate point mutations in MAP4 (S768A and S787A) through polymerase chain reaction reactions using the QuikChange^®^ Multi Site-Directed Mutagenesis Kit, and the MKK6(Glu) adenovirus was constructed as previously described (Hu et al., 2014; Li et al., 2018). Both adenoviruses were constructed by the GeneChem Company (Shanghai, China) and applied according to the manufacturer’s instructions.

### Western Blot Analysis

Cellular extracts were prepared in RIPA lysis buffer (P0013, Beyotime) for Western blotting and centrifuged at 14,000 rpm for 15 min at 4°C. Supernatants were collected and protein concentrations were determined using a Bradford Protein Quantification Kit (500-0205, Bio-Rad Laboratories). Protein extracts were loaded and separated on SDS-PAGE gels and then transferred to PVDF membranes (Millipore). Membranes were incubated with specific primary antibodies overnight at 4°C. Subsequently, membranes were incubated with secondary antibodies and visualized using a ChemiDoc XRS System (Bio-Rad Laboratories). Primary antibodies used for immunoblotting were: anti-phospho-p38 (p-p38), anti-p38, anti-β-actin, and anti-VDAC purchased from Cell Signaling Technology and were diluted 1:1000; anti-phospho-MAP4 (p-MAP4) and anti-p-MAP4(S787) purchased from GL Biochem were diluted 1:1000; anti-GAPDH (1:5000) and anti-α-tubulin (1:1000) purchased from Proteintech; anti-MAP4 (1:100) purchased from Bethyl; and anti-p-MAP4 (S768) (1:1000) purchased from Biolegend. The in-house rabbit polyclonal antibodies against p-MAP4(S787) was raised against the C-terminal 14 amino acids, KVAEKRT(pS)PSKPSSA, and the respective non-phosphorylated peptides, as described and validated in our previous report (Hu et al., 2014; Li et al., 2018). Here, we raised and validated the rabbit polyclonal antibody against p-MAP4 using the amino acids QAKVG(pS)LDNVGHLPAGc, and the respective non-phosphorylated peptides conjugated to bovine serum albumin (BSA) (Supplementary Figure S1).

### Immunofluorescence Staining of MTs

Cells cultured on glass coverslips were fixed with 4% paraformaldehyde for 20 min, permeabilized with 0.1% Triton X-100 in phosphate-buffered saline (PBS) for 25 min, and then blocked with 10% goat serum for 1 h. For the staining of MT structures, a rabbit anti-α-tubulin primary antibody (1:100, Proteintech, United States) was diluted with PBS, and the coverslips were incubated at 4°C overnight. The coverslips were washed with PBS and then incubated with a goat anti-rabbit secondary antibody conjugated to cyanine 3 (Cy3, 1:100) for 1 h at 37°C. The cells were imaged using a confocal microscope (Leica Microsystems, Wetzlar, Germany).

### Cell Proliferation Assay

5-Ethynyl-2′-deoxyuridine (EdU) is a thymidine analog whose incorporation is used to label cells undergoing DNA replication (Salic and Mitchison, 2008). Cell proliferation was determined with the EdU assay using the Click-iT^®^ EdU imaging detection kit, according to the manufacturer’s instructions (BCK488-IV-IM-S, Sigma, United States). The EdU-positive cells are defined as proliferating cells. The fluorescent images were finally obtained using a Leica Confocal Microscope (Leica Microsystems, Germany).

### Extraction and Quantification of Tubulin Fractions

The free and polymerized tubulin fractions were isolated using a previously described method (Putnam et al., 1998). Cells cultured in 6-well plates were washed twice with an MT stabilization buffer (MTSB, 37°C) containing 0.1 M piperazine-*N, N*′-*bis* (2-ethanesulfonic acid, pH 6.8) (PIPES), 2 mM ethylene glycol-*bis* (β-aminoethylether)-*N, N, N*′, *N*′-tetraacetic acid (EGTA), 2 mM ethylenediaminetetraacetic acid (EDTA), 0.5 mM MgCl_2_, and 20% glycerol. Cells were incubated with MTSB + 0.1% Triton X-100 + protease inhibitor cocktail (Sigma-Aldrich) + phenylmethylsulfonyl fluoride (PMSF, Sigma-Aldrich) + phosphatase inhibitor cocktail (Sigma-Aldrich). Western blots were performed to analyze the polymerized and free tubulin fractions.

### Statistical Analysis

All data are presented as the means ± SEM. Comparisons between two groups were performed using a two-tailed unpaired *t*-test. The statistical significance of differences among three or more groups was assessed using a one-way analysis of variance (ANOVA). *P <* 0.05 was considered a significant difference.

## Author Contributions

YH supervised the study. LL and JZ designed the experiments with help from JH and DZ. JZ, LL, and QZ performed the experiments with help from XY, CZ, XZ, HS, BC, and YLi. JZ, LL, and YLv analyzed the data. JZ, LL, and JH cowrote the manuscript. All authors discussed the results and commented on the manuscript.

## Conflict of Interest Statement

The authors declare that the research was conducted in the absence of any commercial or financial relationships that could be construed as a potential conflict of interest.

## Footnotes

1 http://rsb.info.nih.gov/ij/

## References

[B1] Akhmanova A. Steinmetz M. O. (2015). Control of microtubule organization and dynamics: two ends in the limelight. *Nat. Rev. Mol. Cell Biol.* 16 711–726. 10.1038/nrm4084 26562752

[B2] Banyard D. A. Adnani B. O. Melkumyan S. Araniego C. A. Widgerow A. D. (2016). Endothelial progenitor cells and burn injury - exploring the relationship. *Burns Trauma* 4:4. 10.1186/s41038-016-0028-x 27574674 PMC4964096

[B3] Cassimeris L. (2002). The oncoprotein 18/stathmin family of microtubule destabilizers. *Curr. Opin. Cell Biol.* 14 18–24. 10.1016/S0955-0674(01)00289-7 11792540

[B4] Chang W. Gruber D. Chari S. Kitazawa H. Hamazumi Y. Hisanaga S. (2001). Phosphorylation of MAP4 affects microtubule properties and cell cycle progression. *J. Cell Sci.* 114 2879–2887. 11683421 10.1242/jcs.114.15.2879

[B5] Chen X. Zhang S. Wang Z. Wang F. Cao X. Wu Q. (2018). Supervillin promotes epithelial-mesenchymal transition and metastasis of hepatocellular carcinoma in hypoxia via activation of the RhoA/ROCK-ERK/p38 pathway. *J. Exp. Clin. Cancer Res.* 37:128. 10.1186/s13046-018-0787-2 29954442 PMC6025706

[B6] DiPietro L. A. (2013). Angiogenesis and scar formation in healing wounds. *Curr. Opin. Rheumatol.* 25 87–91. 10.1097/BOR.0b013e32835b13b6 23114588

[B7] Falanga V. (1988). Occlusive wound dressings. Why, when, which?. *Arch. Dermatol.* 124 872–877. 10.1001/archderm.1988.01670060018009 3288123

[B8] Giampietro C. Luzzati F. Gambarotta G. Giacobini P. Boda E. Fasolo A. (2005). Stathmin expression modulates migratory properties of GN-11 neurons in vitro. *Endocrinology* 146 1825–1834. 10.1210/en.2004-0972 15625246

[B9] Hirota Y. Yamashita S. Kurihara Y. Jin X. Aihara M. Saigusa T. (2015). Mitophagy is primarily due to alternative autophagy and requires the MAPK1 and MAPK14 signaling pathways. *Autophagy* 11 332–343. 10.1080/15548627.2015.1023047 25831013 PMC4502654

[B10] Hopf H. W. Rollins M. D. (2007). Wounds: an overview of the role of oxygen. *Antioxid. Redox Signal.* 9 1183–1192. 10.1089/ars.2007.1641 17536961

[B11] Hu J. Chu Z. Han J. Zhang Q. Zhang D. Dang Y. (2014). Phosphorylation-dependent mitochondrial translocation of MAP4 is an early step in hypoxia-induced apoptosis in cardiomyocytes. *Cell Death Dis.* 5:e1424. 10.1038/cddis.2014.369 25232678 PMC4540186

[B12] Hu J. Y. Chu Z. G. Han J. Dang Y. M. Yan H. Zhang Q. (2010). The p38/MAPK pathway regulates microtubule polymerization through phosphorylation of MAP4 and Op18 in hypoxic cells. *Cell. Mol. Life Sci.* 67 321–333. 10.1007/s00018-009-0187-z 19915797 PMC11115776

[B13] Huang C. Jacobson K. Schaller M. D. (2004). MAP kinases and cell migration. *J. Cell Sci.* 117(Pt 20), 4619–4628. 10.1242/jcs.01481 15371522

[B14] Jiang Y. Y. Shang L. Shi Z. Z. Zhang T. T. Ma S. Lu C. C. (2016). Microtubule-associated protein 4 is an important regulator of cell invasion/migration and a potential therapeutic target in esophageal squamous cell carcinoma. *Oncogene* 35 4846–4856. 10.1038/onc.2016.17 26876215

[B15] Joseph J. V. Conroy S. Pavlov K. Sontakke P. Tomar T. Eggens-Meijer E. (2015). Hypoxia enhances migration and invasion in glioblastoma by promoting a mesenchymal shift mediated by the HIF1alpha-ZEB1 axis. *Cancer Lett.* 359 107–116. 10.1016/j.canlet.2015.01.010 25592037

[B16] Kaverina I. Straube A. (2011). Regulation of cell migration by dynamic microtubules. *Semin. Cell Dev. Biol.* 22 968–974. 10.1016/j.semcdb.2011.09.017 22001384 PMC3256984

[B17] Kim E. K. Choi E. J. (2010). Pathological roles of MAPK signaling pathways in human diseases. *Biochim. Biophys. Acta* 1802 396–405. 10.1016/j.bbadis.2009.12.009 20079433

[B18] Kitazawa H. Iida J. Uchida A. Haino-Fukushima K. Itoh T. J. Hotani H. (2000). Ser787 in the proline-rich region of human MAP4 is a critical phosphorylation site that reduces its activity to promote tubulin polymerization. *Cell Struct. Funct.* 25 33–39. 10.1247/csf.25.33 10791892

[B19] Kreitzer G. Myat M. M. (2018). Microtubule motors in establishment of epithelial cell polarity. *Cold Spring Harb. Perspect. Biol.* 10:a027896. 10.1101/cshperspect.a027896 28264820 PMC5793753

[B20] Kremer B. E. Haystead T. Macara I. G. (2005). Mammalian septins regulate microtubule stability through interaction with the microtubule-binding protein MAP4. *Mol. Biol. Cell* 16 4648–4659. 10.1091/mbc.e05-03-0267 16093351 PMC1237071

[B21] LaFlamme S. E. Mathew-Steiner S. Singh N. Colello-Borges D. Nieves B. (2018). Integrin and microtubule crosstalk in the regulation of cellular processes. *Cell. Mol. Life Sci.* 75 4177–4185. 10.1007/s00018-018-2913-x 30206641 PMC6182340

[B22] Lamalice L. Le Boeuf F. Huot J. (2007). Endothelial cell migration during angiogenesis. *Circ. Res.* 100 782–794. 10.1161/01.RES.0000259593.07661.1e 17395884

[B23] Li L. Hu J. He T. Zhang Q. Yang X. Lan X. (2015). P38/MAPK contributes to endothelial barrier dysfunction via MAP4 phosphorylation-dependent microtubule disassembly in inflammation-induced acute lung injury. *Sci. Rep.* 5:8895. 10.1038/srep08895 25746230 PMC4352893

[B24] Li L. Zhang Q. Zhang X. Zhang J. Wang X. Ren J. (2018). Microtubule associated protein 4 phosphorylation leads to pathological cardiac remodeling in mice. *EBioMedicine* 37 221–235. 10.1016/j.ebiom.2018.10.017 30327268 PMC6286641

[B25] Li N. Jiang P. Du W. Wu Z. Li C. Qiao M. (2011). Siva1 suppresses epithelial-mesenchymal transition and metastasis of tumor cells by inhibiting stathmin and stabilizing microtubules. *Proc. Natl. Acad. Sci. U.S.A.* 108 12851–12856. 10.1073/pnas.1017372108 21768358 PMC3150944

[B26] Liu B. P. Chrzanowska-Wodnicka M. Burridge K. (1998). Microtubule depolymerization induces stress fibers, focal adhesions, and DNA synthesis via the GTP-binding protein Rho. *Cell Adhes Commun.* 5 249–255. 10.3109/15419069809040295 9762466

[B27] Munoz-Chapuli R. Quesada A. R. Angel Medina M. (2004). Angiogenesis and signal transduction in endothelial cells. *Cell. Mol. Life Sci.* 61 2224–2243. 10.1007/s00018-004-4070-7 15338053 PMC11138771

[B28] Nagaraju G. P. Bramhachari P. V. Raghu G. El-Rayes B. F. (2015). Hypoxia inducible factor-1alpha: its role in colorectal carcinogenesis and metastasis. *Cancer Lett.* 366 11–18. 10.1016/j.canlet.2015.06.005 26116902

[B29] Ookata K. Hisanaga S. Bulinski J. C. Murofushi H. Aizawa H. Itoh T. J. (1995). Cyclin B interaction with microtubule-associated protein 4 (MAP4) targets p34cdc2 kinase to microtubules and is a potential regulator of M-phase microtubule dynamics. *J. Cell Biol.* 128 849–862. 10.1083/jcb.128.5.849 7876309 PMC2120387

[B30] O’Toole E. A. Marinkovich M. P. Peavey C. L. Amieva M. R. Furthmayr H. Mustoe T. A. (1997). Hypoxia increases human keratinocyte motility on connective tissue. *J. Clin. Invest.* 100 2881–2891. 10.1172/JCI119837 9389755 PMC508495

[B31] Ou Y. Zheng X. Gao Y. Shu M. Leng T. Li Y. (2014). Activation of cyclic AMP/PKA pathway inhibits bladder cancer cell invasion by targeting MAP4-dependent microtubule dynamics. *Urol. Oncol.* 32 e21–e28. 10.1016/j.urolonc.2013.06.017 24140250

[B32] Park E. C. Rongo C. (2016). The p38 MAP kinase pathway modulates the hypoxia response and glutamate receptor trafficking in aging neurons. *eLife* 5:e12010. 10.7554/eLife.12010 26731517 PMC4775213

[B33] Parysek L. M. Asnes C. F. Olmsted J. B. (1984). MAP 4: occurrence in mouse tissues. *J. Cell Biol.* 99(4 Pt 1), 1309–1315. 10.1083/jcb.99.4.1309 6384236 PMC2113335

[B34] Putnam A. J. Cunningham J. J. Dennis R. G. Linderman J. J. Mooney D. J. (1998). Microtubule assembly is regulated by externally applied strain in cultured smooth muscle cells. *J. Cell Sci.* 111(Pt 22), 3379–3387. 9788879 10.1242/jcs.111.22.3379

[B35] Ramkumar A. Jong B. Y. Ori-McKenney K. M. (2018). ReMAPping the microtubule landscape: how phosphorylation dictates the activities of microtubule-associated proteins. *Dev. Dyn.* 247 138–155. 10.1002/dvdy.24599 28980356 PMC5739964

[B36] Salic A. Mitchison T. J. (2008). A chemical method for fast and sensitive detection of DNA synthesis in vivo. *Proc. Natl. Acad. Sci. U.S.A.* 105 2415–2420. 10.1073/pnas.0712168105 18272492 PMC2268151

[B37] Srsen V. Kitazawa H. Sugita M. Murofushi H. Bulinski J. C. Kishimoto T. (1999). Serum-dependent phosphorylation of human MAP4 at Ser696 in cultured mammalian cells. *Cell Struct. Funct.* 24 321–327. 10.1247/csf.24.321 15216889

[B38] Steinmetz M. O. Prota A. E. (2018). Microtubule-targeting agents: strategies to hijack the cytoskeleton. *Trends Cell Biol.* 28 776–792. 10.1016/j.tcb.2018.05.001 29871823

[B39] Stramucci L. Pranteda A. Bossi G. (2018). Insights of crosstalk between p53 protein and the MKK3/MKK6/p38 MAPK signaling pathway in cancer. *Cancer* 10:E131. 10.3390/cancers10050131 29751559 PMC5977104

[B40] Xia X. He C. Wu A. Zhou J. Wu J. (2018). Microtubule-associated protein 4 is a prognostic factor and promotes tumor progression in lung adenocarcinoma. *Dis. Mark.* 2018:8956072. 10.1155/2018/8956072 29743960 PMC5878896

[B41] Xing D. Liu L. Marti G. P. Zhang X. Reinblatt M. Milner S. M. (2011). Hypoxia and hypoxia-inducible factor in the burn wound. *Wound Repair Regenerat.* 19 205–213. 10.1111/j.1524-475X.2010.00656.x 21362088 PMC3075089

[B42] Yip W. L. (2015). Influence of oxygen on wound healing. *Int. Wound J.* 12 620–624. 10.1111/iwj.12324 24974913 PMC7950466

[B43] You J. Wang Z. Xu S. Zhang W. Fang Q. Liu H. (2016). Advanced glycation end products impair glucose-stimulated insulin secretion of a pancreatic beta-cell line INS-1-3 by disturbance of microtubule cytoskeleton via p38/MAPK activation. *J. Diabetes Res.* 2016:9073037. 10.1155/2016/9073037 27635403 PMC5011238

[B44] Zhang J. Zhang C. Jiang X. Li L. Zhang D. Tang D. (2019). Involvement of autophagy in hypoxia-BNIP3 signaling to promote epidermal keratinocyte migration. *Cell Death Dis.* 10:234. 10.1038/s41419-019-1473-9 30850584 PMC6408485

[B45] Zhang J. Zhang D. Yan T. Jiang X. Zhang C. Zhao L. (2017). BNIP3 promotes the motility and migration of keratinocyte under hypoxia. *Exp. Dermatol.* 26 416–422. 10.1111/exd.13248 27783443

