# Supplementary material for: Phosphorylation of Microtubule- Associated Protein 4 Promotes Hypoxic Endothelial Cell Migration and Proliferation
Source: Front Pharmacol. 2019 Apr 16;10:368. doi: 10.3389/fphar.2019.00368 (PMC6476958; doi:10.3389/fphar.2019.00368)

# Supplementary material

## Microtubule associated protein 4 promotes hypoxic endothelial cells migration and proliferation through its phosphorylation

Junhui Zhang[1,2*], Lingfei Li[1,2*], Qiong Zhang[1,2], Xu Yang[3], Can Zhang[4], Xingyue Zhang[1,2], Dongxia Zhang[1,2], Yanling Lv[1,2], Huapei Song[1,2], Bing Chen[5], Yao Liu[6], Jiongyu Hu[2,5*], Yuesheng Huang[1,2*]

[1]Institute of Burn Research, Southwest Hospital, Third Military Medical University (Army Medical University), Chongqing, China.

[2]State Key Laboratory of Trauma, Burns and Combined Injury, Southwest Hospital, Third Military Medical University (Army Medical University), Chongqing, China.

[3]Department of Respiratory Medicine. The 983 Hospital of Joint Logistics Support Force of the Chinese People's Liberation Army, Tianjin, China.

[4]Department of Plastic Surgery, Southwest Hospital, Army Medical University (Third Military Medical University), Chongqing, China.

[5]Endocrinology Department, Southwest Hospital, Third Military Medical University (Army Medical University), Chongqing, China.

[6]Department of Pharmacy, Southwest Hospital, Third Military Medical University (Army Medical University), Chongqing, China.

Corresponding authors:

Yuesheng Huang, Institute of Burn Research, State Key Laboratory of Trauma, Burns and Combined Injury, Southwest Hospital, Third Military Medical University (Army Medical University), Gaotanyan Street, Shapingba District, Chongqing 400038, China. Phone: +86 023 68766023. E-mail: yshuang1958@163.com;

Jiongyu Hu, Endocrinology Department, State Key Laboratory of Trauma, Burns and Combined Injury, Southwest Hospital, Third Military Medical University (Army Medical University), Gaotanyan Street, Shapingba District, Chongqing 400038, China. Phone: +86 023 68773162. E-mail: jiongyuhu@163.com.

[*]These authors contributed equally to this work.

**Figure S1. Validation of antibody made in-house.** Preparation and verification of anti-p-MAP4 polyclonal antibodies by Dot blot. Np-pep: non-phospho-peptide, p-pep: phosphor-peptide, Np-Ab: non-phospho-antibody, p-Ab: phosphor-antibody.

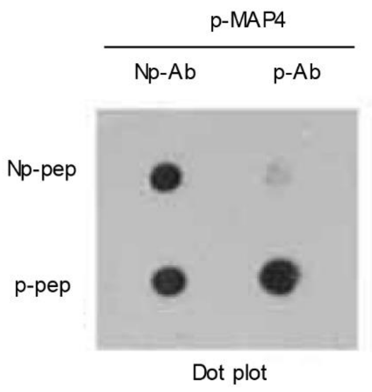

Supplement: Supplementary file 1 [file Data_Sheet_1.PDF]
